# The Benefits of Being a “Buddy”: Exploring the Medical Student Experience As Mentor to Minority High-School Students

**DOI:** 10.1089/heq.2020.0060

**Published:** 2021-01-11

**Authors:** Rosellen Roche, Joel Manzi, Bradley C. Kruithoff

**Affiliations:** Department of Primary Care, Ohio University College of Osteopathic Medicine, Cleveland, Ohio, USA.

**Keywords:** medical education, mentorships, medical students, volunteerism, community medicine

## Abstract

**Purpose:** *The Aspiring Doctors Precollege Program* at Ohio University Heritage College of Osteopathic Medicine serves to introduce underrepresented minority (URM) high-school students to careers in health care as well as introducing URM high-school students to medical student mentors. Each month, medical students and their student mentees connect through a variety of activities on the medical college campus. While the program has significant benefit for the mentees, it also provides professional development opportunities for the medical students as mentors. Many researchers have written on the value of mentored relationships between medical students and established physicians; however, exploring the benefits of medical student mentorship has yet to be discussed in the literature.

**Objectives:** The primary objectives of this study are to understand medical student perceptions of being a mentor and describe the contributions to their medical education.

**Methods:** Semistructured interviews were conducted with student mentors regarding their experiences serving in this program. These interviews were inductively coded for significant ideas, themes, and patterns.

**Results:** A series of 12 research interviews were conducted with medical students who have participated in *The Aspiring Doctors* program for at least three semesters. Major themes that emerged from the analysis include the following: the importance of guidance in medicine through person-to-person mentoring, and identification of future career aspirations.

**Summary/Conclusions:** Medical student mentors found this program to be a valuable addition to their educational experience. Mentoring URM high-school students offers pre-clinical medical students the opportunity to connect with their community and envision themselves serving as physicians in underresourced communities. Simultaneously, it provides a meaningful way of paying-it-forward during their education. Further studies can be done to track the outcomes of the medical students with respect to their designations stated while participating in this program, the role of mentorship on professional identity development, and possible effects on preventing/mitigating burnout.

## Introduction

Diversity is a heralded component of medical school mission statements across the United States. Despite this, there continues to be a lack of substantial diversity in the physician workforce. Although representing 39.6% of the US population, only 12.9% of physicians identify with a group that is underrepresented in the physician workforce.^1,2^ However, underrepresented minority (URM) physicians play a crucial role in health care delivery. Physicians of African American, Native American, or Hispanic origin are more likely to practice in medically underserved areas than Caucasian physicians.^3–7^ While there has been an increase in minority medical school enrollment, URM students represent 28% of the entering medical school class of 2019.^2^ There are myriad barriers to entry into medicine that exist for URM candidates.^8–12^ One such barrier is a lack of mentorship opportunities connecting students and URM medical professionals to assist in navigating the complicated educational and application processes.

To help students counter these barriers, the Ohio University Heritage College of Osteopathic Medicine (OU-HCOM)—Cleveland, founded *The Aspiring Doctors Precollege Program* for high achieving URM high-school students. Students complete a year-long program at the medical school campus that prepares them for success in college and, eventually, medical school. Through this experience, students have an opportunity to learn clinical skills, participate in hands-on laboratory activities, and gain college preparatory guidance. In addition to these learning experiences, another foundational aspect of this program comes from the pairing of each student with a current medical student mentor. These medical student mentors (“buddies”) participate in a variety of activities with their students throughout the program.

There has been much discussion in the educational literature on the benefits of medical students receiving mentorship from attending physicians and residents.^13–15^ These studies have shown that mentorship programs are an effective means of promoting medical student advancement into specific specialties and facilitating community engagement.^8,16–18^ While these benefits are commendable, there has been little discussion about the capacity of medical students to serve as mentors, and importantly, the value that providing mentorship can add to the medical students' educational experience.^19,20^

The purpose of this study is to query medical students regarding their experiences as mentors, and to explore the potential effects this could have on their future careers. Through this investigation, important themes emerged from the medical student experience, namely that this experience confirmed the importance of providing guidance through one-on-one relationships, and confirmed future career aspirations.

## Methods

### Participants

Since 2016 there have been a total of 58 medical student mentors involved in the program. Mentors volunteer for a minimum commitment of 1 year during either their first or second year of medical school. If students elect to continue in the program, they will be paired with the same high-school students to promote continuity in the relationship.

For this study, medical student mentors were recruited via email to participate in research interviews regarding their experiences in the program. These interviews were limited to students who had volunteered for a minimum of three semesters. This limit was established to focus on the long-term impacts of participation in this program. This also allowed for commentary and reflection on the impacts noted during the clinical stages of medical education.

### Data collection and analysis

Medical student mentors were interviewed in a semistructured format consisting of a question script regarding their experience in the program, their desire to become mentors, and the impact this program had on their professional career aspirations.^21^ Interviews began with this open-ended question script, but conversations were allowed to flow naturally depending on the answers of the respondent, while still covering key topics and areas of concern.^22^ Each interview lasted between 15 and 25 min. Participants were compensated with a gift card in exchange for participation.

**FIG. 1.**
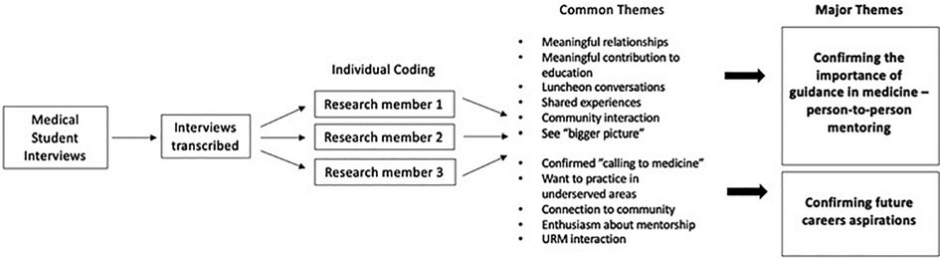
Flowchart describing the process of inductive coding from the semistructured interviews of the medical student Aspiring Doctors participants.

The interviews were transcribed by a third-party transcription service and returned to the research team. The transcripts were analyzed independently by research team members through inductive analysis to look for patterns, resemblances, and regularities in experiences.^22^ Each researcher created a coded framework of the transcribed interviews. The researchers then met to review their individual coded findings and determine the strongest themes identified by all researchers.^23^ Any discrepancies in coding were resolved by group consensus and harmonized into the qualitative themes as described in the Results section. A brief overview of this process can be found in Figure 1.

Ohio University Institutional Review Board approved this project.

### Results

A total of 12 mentors were interviewed for this project. Mentors were selected from current second- or third-year medical students who had been mentors for at least three semesters. Half of the mentors interviewed identified as URM, and 10 (83.3%) of the mentors interviewed were women. An overview of the demographic information of the mentors can be found in Table 1.

**Table 1.** Characteristics of Medical Student Mentors Interviewed

Student characteristics	Mentors, N (%)
Gender	
Male	2 (16.7)
Female	10 (83.3)
Rank	
OMS-II	4 (33.3)
OMS-III	8 (66.7)
URM	
Self-identify URM	6 (50)
Does not self-identify URM	6 (50)
Medical specialty designation	
Primary care	8 (66.7)
Nonprimary care	2 (16.7)
Undecided	2 (16.7)

OMS, osteopathic medical student; URM, underrepresented minority.

### The importance of providing guidance: person-to-person mentoring

As intended by the program objectives, the mentors and students formed meaningful relationships. This was the most commonly cited theme by all the interviewed mentors. Through this program, authentic relationships were organically formed during formal and informal activities. In these activities, medical students had the opportunity to hone their mentorship ability and give advice to their mentees on a variety of topics. While these activities are not normal parts of pre-clinical medical education, all mentors agreed that this experience contributed greatly to their educational experience. As medical education follows an apprenticeship model, with attending physicians guiding residents, and residents guiding medical students, students recognized that they could also serve in this apprenticeship model and impart useful information to rising students.

### Connecting around free-time, food, and conversation

On-campus lunches were provided for all participants of the program. During this informal break between activities, mentors and students could relax and allow conversations to flow naturally. All of the mentors discussed the power in these unscripted interactions in the development of authentic relationships.

The luncheons. I think those were great. I really got to know them and got to know them as people, and their interests, and their families. I think that alone was really meaningful because they were comfortable asking me things, and taking my advice, and directing their future interests based on my advice and opinions.

Medical students commented on their ability to connect with the URM students on formal academic issues such as advice and college planning; and also on an informal level regarding shared interests. In sum, the informal atmosphere of the lunch time was a great bonding opportunity for the mentors. The medical students felt they were able to give open and honest responses to the student's questions.

We have lunch with them. We'll all go off in a separate corner of the room and catch up with them, and ask them how things are going… if they've completed their SAT, ACT. What do they do to follow up related to applying to colleges, and any interviews that they're getting, etc.

I just loved connecting with them, talking with them. [I]n the in-between time is when I had the most fun, just relating to them, talking to them about things they like to do, shows they like to watch. And we actually had a lot in common!

### Giving advice and guidance

In these informal situations, mentors appreciated the ability to give advice to their mentees. Sharing their experiences in medical school and offering suggestions to students just starting their educational career were meaningful for these mentors.

I think it was helpful to have somebody that they didn't really know, that wasn't their family, that they could confide in, it was an unbiased opinion. And to get advice from somebody from that kind of perspective, was helpful for them. Kind of along the lines of being the big sister and giving them advice.

So, I think just talking general life… how to manage the college application process, and taking the tests, and studying, and how you're managing having a job, and how you decide what you want to do and what school to go to. Just kind of all of that, just the stepping-stones from going from high school to college and thinking about what you want to do. I think it was helpful for them to realize that there's options for anything they want to do.

### A meaningful addition to the medical curriculum

The medical students commented on the positive addition serving as a mentor had on their pre-clinical education. Traditional pre-clinical medical education is academically rigorous. Serving as a mentor allows the medical students the opportunity to interact with members of the community, and allows a break from their studies. The medical students commented that their time as a mentor was constructive and did not negatively take away from their coursework. Rather, it positively complemented their course work and overall experience.

“[This program] gave me something to do outside of just studying all day. Something to look forward to. When I know they are coming to campus, it's exciting.

It helped me; I would say especially second year. Whenever things were kind of heating up, boards, it helped me, basically made me, take a break those days that they were coming and do something to give back, which is something I always have liked, have always enjoyed.

Several of the medical students noted the relationships they forged with the students were similar to the relationships they had developed in their medical education. Students in the clinical stages of education noted that practicing this mentorship role helped them to see the larger benefit of mentorship in their own education.

I believe this prepared me for the apprenticeship approach to medicine that has existed for many years. We're about to be residents and we're professional learners for the rest of our lives, and we're about to be interns learning from residents, and residents learning from attendings, and attendings learning from peers. I think this was laying a good groundwork.

I think that in every chapter of our education, we will interact with people higher and lower than us in age and rank. One of the biggest things that I took away from this is that you can be mentored by a younger person. I just think that a lot of people aren't willing to admit that young people can teach you things. I learned a lot from them, just as much as they did from me.

### Confirming future career aspirations

Many students upon admission to medical school express a desire to work with underserved populations. In these interviews, students stated that becoming a mentor solidified these plans in a positive way. Many of the medical student mentors identified interests in primary care, practicing in underserved communities, and serving as a formal mentor in their future career.

#### Confirmation of a calling to medicine

Several of the buddies recognized that participating in this program caused them to remember why they entered the medical field and established a sense of confirmation of their chosen career path.

“It also made me really thankful that I like medicine so much… and being able to educate someone on it, really it's just a growing experience I think is really improved my relationship with medicine because I'm not as negative about it.

I'm actually interested in going to Pediatrics, and I value the youth. I value just building up the next generation and for me, mentorship will always be a part of my career, no matter what I'm doing, no matter where I'm at, I always plan to create mentorship opportunities.

#### Connections to background and home communities

While not all of the mentors identified as URM, those who did identified a connection with their home communities. Giving back to their communities was a very important aspect of practicing medicine, specifically primary care. This program allowed them to model an aspect of community leadership in their medical education.

It's definitely confirmed that I've always been interested in family medicine, working in urban underserved communities with minority communities. Communities that I've lived in and been a part of my whole life, going back and serving them. Really it gives me an opportunity to act on that interest. I think this program has definitely confirmed and continued to reinforce that interest.

I do actually want to teach long-term and also work in urban communities. I know family medicine will allow me to work with multiple age groups. It's rewarding for me, coming from an urban background myself, and I hope it's beneficial for them.

#### Becoming mentors who make a difference

Many of the medical students identified that they currently have physician mentors who inspired them to serve as mentors themselves. Many of the medical students who self-identified as URM stated that working with the URM population initially encouraged them to join the program:

I feel like I've benefited from the relationship because it means a lot to me to have interactions with young women of color and trying to help them out because I've been there.

It's vital to my success as a minority student to have individuals who do look like me as I go through this journey. It's been very, very huge… That's why I want to do the same thing for students. I understand the importance of having minority physicians or medical students guiding other minorities through this journey.

A huge part of why I wanted to become a physician is not only caring for patients and seeing them through difficult times in their life, but also advocating for patients and for people in general. I think it gives me an opportunity to really advocate for other students and show them ways that they can advocate for themselves as a high school student.

#### The opportunity to invest early in the community

Many major medical centers are located in urban areas; however, the engagement with the local communities can be limited. This is unfortunately the same with many medical schools. The mentors repeatedly emphasized the importance of hosting this program in this community specifically because of the history of marginalization and disinvestment in the public school systems.

I think this is a really good program to bring more opportunities to the community to see what the school is, what the school is about, and to lend at least our resources as mentors to these students.

I think it's important being that we are in [this community] which is 96% black people. Being able to show, hey, we're here to support the students, we're here to support the community in various ways in terms of health but also in terms of mentorship so we can have students that are helping their community in the future is really powerful.

#### Translation to future career

All of the medical students unanimously agreed that serving as a mentor in *The Aspiring Doctors* program made them want to continue serving as a mentor throughout their professional careers.

I definitely see myself wanting to be at an academic center so I can be exposed to not only colleagues of the same status or position as me but also the next group up and give them a helping hand. And I always keep this mentality in my mind to always help that next group up and not just get stuck with my own career planning.

If something so small as a mentor being able to share with you that you can, then that's awesome. I think paying it forward for sure is awesome and I would continue to do it over and over again, and encourage people to do the same.

## Discussion

The major themes uncovered from our analysis are attributed to the structure of the *Aspiring Doctors* program. Our interpretation of the interviews reflects that involvement in the program comes from their sincere desire to give back to the next generation of medical students.

Many of the medical students stated that their shared experiences with the URM students provided passion for involvement in the program. Many of the medical students come from similarly underserved backgrounds, and viewed it as their duty to “pay-it-forward” to the next generation of students who stand where they once stood. There was a strong theme to provide representation to students of successful and competent URM and/or female medical students. The notion of being the example was echoed throughout the interviews.

Medical students stated that the informal lunch hour was where they were able to refine their interpersonal mentorship skills and communicate with the students. There was an understanding that medical students, although in professional school, were students too, making it easier to connect than with a practicing physician. The medical students stated that they were able to better understand their students during these conversations, and reflect on their shared experiences to foster constructive mentorship. Medical students reflected on their experience as a mentee, and used their personal experiences to complement their switched roll as the mentor. Mentors appreciated that students shared their same aspirations, and were able to offer their opinions on the challenging journey to medical school.

By allowing medical students to model the mentor role early in their medical education, a sense of professional identity was developed that enabled students to see themselves as future physicians. This program provided the chance to encourage and shape self-perceptions and future career goals at a critical time in their education. The idea of serving as a mentor was intertwined with the concept of doctoring from an early stage of their education, prompting many to reconnect and reinforce the underlying convictions that caused them to enter the medical field.

While this program was only implemented at one school, the implications of these findings are significant in their own right. As students have relayed the value this opportunity has added to their educational experience, more training programs should seek to integrate mentorship activities into medical education. With more programs implemented across the country, the long-term benefits of this approach will become evident.

## Conclusion

This research shows that it is formative for medical students to have the opportunity to engage in mentoring activities early in their training. By sitting in both seats of the apprenticeship model, medical students were able to reflect on their own experiences and glean meaningful lessons contributing to a well-rounded education. As described by the mentors themselves, this opportunity identified the importance of one-on-one guidance in navigating medical education, and also reinforced the career aspirations that many students cited as their motivation for entering the medical profession. Through this process, medical students were able to develop a sense of professional identity early in their educational trajectories, which inspired visions of their future careers. The benefit of mentorship not only benefits the URM students of the *Aspiring Doctors* program but is also an investment in the intellectual capital and capacity of future physicians and leaders. Although we have established the positive benefits of mentorship, it will be important to explore the outcomes of medical students as they continue into residency, how their preprofessional experiences contribute to their residency placements, and what effect, if any, these experiences can have on mitigating burnout in postgraduate training.

## Abbreviation Used

URM: underrepresented minority
